# Effects of Sodium-Glucose Cotransporter 2 Inhibition on Glucose Metabolism, Liver Function, Ascites, and Hemodynamics in a Mouse Model of Nonalcoholic Steatohepatitis and Type 2 Diabetes

**DOI:** 10.1155/2020/1682904

**Published:** 2020-12-27

**Authors:** Koichi Yabiku, Keiko Nakamoto, Maho Tsubakimoto

**Affiliations:** ^1^University of the Ryukyus, Division of Endocrinology, Diabetes and Metabolism, Hematology, Rheumatology (Second Department of Internal Medicine), Graduate School of Medicine, Okinawa, Japan; ^2^GenomIdea Incorporated, Okinawa, Japan; ^3^Department of Radiology, Graduate School of Medical Science, University of the Ryukyus, Okinawa, Japan

## Abstract

Many blood glucose-lowering drugs cannot be used once patients with type 2 diabetes (T2D) and nonalcoholic fatty liver disease develop nonalcoholic steatohepatitis (NASH). Therefore, such patients often require insulin treatment. We aimed to determine the effect of sodium-glucose cotransporter 2 inhibitor (SGLT2i) dapagliflozin monotherapy on glucose metabolism in a mouse model of NASH/T2D, with a focus on its diuretic effects. To imitate ascites and to determine its severity by imaging, meglumine sodium amidotrizoate (MSA) was infused into the abdominal cavities of mice. The reduction in ascites induced by dapagliflozin was compared with that induced by furosemide using microcomputed tomography. The effects of each drug on hemodynamics were also compared. A dapagliflozin-related improvement in glucose tolerance was achieved in mice fed a high-fat diet (HFD) or an HFD + methionine-and-choline-deficient diet (MCDD). In dapagliflozin-treated NASH mice, hypoglycemia was not identified during 24-hour casual blood glucose monitoring. In the dapagliflozin and furosemide-treated groups, the time taken for the resolution of artificial ascites was significantly shorter than in the untreated group, and there were no significant differences between these groups. Furosemide significantly reduced the blood pressure and significantly increased the heart rate of the mice. Dapagliflozin caused a mild decrease in systolic, but not diastolic blood pressure, and the heart rate and circulating catecholamine and renin-aldosterone concentrations were unaffected. Dapagliflozin treatment improved glycemic control in the NASH mice versus untreated mice. Thus, dapagliflozin had a prompt diuretic effect but did not adversely affect the hemodynamics of mice with NASH and T2D. Therefore, it may be useful for the treatment of patients with both T2D and liver cirrhosis.

## 1. Introduction

Sodium-glucose cotransporter 2 (SGLT2) is expressed in the proximal kidney tubule, where it mediates the reabsorption of approximately 90% of the glucose filtered through the glomerulus [1, 2]. Selective SGLT2 inhibitors (SGLT2is) promote the urinary excretion of glucose, thereby having an antihyperglycemic effect [3]. Furthermore, a previous study has shown that these agents also reduce body mass and blood pressure, and improve lipid and uric acid metabolism [4]. Visible effects of these drugs, such as weight loss, improve treatment compliance, which maximizes the other effects of the drug [5]. Additionally, it has been shown that SGLT2i has renoprotective effects by ameliorating excessive glomerular filtration [6]. These pleiotropic effects of SGLT2i were shown to significantly reduce the incidences of cardiovascular mortality and heart failure in patients at high risk of cardiovascular events versus placebo [7]. In the LEADER study, a glucagon-like peptide- (GLP-) 1 receptor agonist, liraglutide, significantly inhibited cardiovascular events in patients with T2D between 2 and 3 years after the start of administration [8]. However, the EMPA-REG OUTCOME study showed that a positive effect on the incidence of cardiovascular death is achieved only 1 year after the start of administration of an SGLT2i [7].

Some previous studies have shown that treatment with an SGLT2i is associated with increases in blood transaminase activities [4, 9]. However, there have been few studies of the clinical efficacy or safety of the use of SGLT2i in patients with high liver enzyme activities. Liver function deteriorates in many patients with diabetes, and the incidence of fatty liver among these patients is high [10], which has a significant negative impact upon glycemic control. Nonalcoholic fatty liver disease (NAFLD), which frequently develops in the presence of metabolic syndrome, has been investigated as a possible etiological factor for T2D, given that both are characterized by insulin resistance [11–16]. In contrast to the effects of drugs that are conventionally used for the treatment of T2D, SGLT2is ameliorate insulin resistance by reducing visceral fat mass; therefore, they may also be effective for the treatment of patients with both T2D NAFLD.

However, approximately 20%–30% of patients with NAFLD may progress to nonalcoholic steatohepatitis (NASH) within 5 years [17, 18]. When patients with T2D and NAFLD progress to NASH, the use of most blood glucose-lowering drugs is restricted; therefore, treatment with insulin is often required. Glycogen cannot accumulate in a fibrotic liver and glycogenesis is inadequate, such that both hepatic insulin metabolism and glucose disposal are limited. Therefore, unexpected postprandial hyperglycemia or fasting/nocturnal hypoglycemia may occur, despite careful therapeutic planning.

Notably, in the presence of ascites, nutrient balance is affected, with a reduction in appetite that complicates glycemic control. However, there have been no studies of the effects of SGLT2is in patients with or models of advanced liver cirrhosis with ascites. In the present study, we aimed to determine whether the SGLT2i dapagliflozin has antihyperglycemic, urinary glucose excretion-promoting, and diuretic effects in a mouse model of diabetes and NASH. In particular, we evaluated the effects of dapagliflozin on the blood glucose concentration, ascites, and hemodynamics of the mice.

## 2. Materials and Methods

### 2.1. Animal and Experimental Protocols

Male 5-week-old C57BL/6J mice were purchased from the Jackson Laboratory (CA, USA). Mice were housed (*n* = 5 per cage) in a temperature-controlled room (22–23°C) under a 12 h light/dark cycle. Animal experiments were approved by the Institutional Animal Care Committee of the Faculty of Medicine at the University of the Ryukyus (No. A2016112), and followed the ARRIVE guidelines.

**Figure 1:**
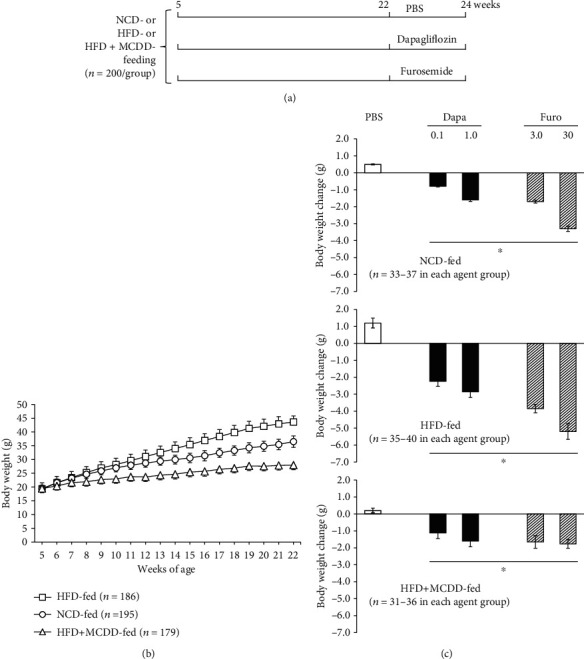
The body mass of the HFD-fed group increased more than that of the NCD-fed group, and the HFD + MCDD-fed group gained little. (a) Experimental design (*n* = 200 per diet group). (b) Body mass change in each diet group (*n* = 179‐195 per group). (c) Body mass change during 2 weeks of treatment with each agent. Comparison between mice that were administered phosphate-buffered saline (PBS) or 0.1 or 1.0 mg/kg/day dapagliflozin (Dapa) or 3.0 or 30 mg/kg/day furosemide (Furo). Data are means ± SEMs. ^∗^*P* < 0.01*vs.* PBS-treated mice in each diet group for the change during the 2 weeks of treatment.

Figure 1(a) shows the experimental design. The mice were randomized to three groups on the basis of their body mass: one was fed a normal chow diet (NCD; 5% fat) (CE-2, CLEA Japan Inc., Tokyo, Japan), the second was fed a high-fat diet (HFD; 56% of calories from fat) (F2HFD2, Oriental Yeast Co., Ltd., Tokyo, Japan), and the third was fed a high-fat, methionine-and-choline-deficient diet (HFD + MCDD; 21% of calories from fat) (A02082002BR, Research Diets, Inc. USA) from 5 to 24 weeks of age. Groups of mice were administered vehicle (phosphate-buffered saline (PBS)), dapagliflozin (Dapa) at 0.1 or 1.0 mg/kg/day, or furosemide (Furo) at 3.0 or 30 mg/kg/day during the last 2 weeks of the feeding period (*n* = 40 per group). To evaluate liver damage, glucose tolerance, and hormone concentrations, blood samples (approximately 15 *μ*l) were collected in the fasting state from a tail vein using capillary glass tubes before and after the period of administration of each agent.

### 2.2. Measurement of Plasma Transaminase Activities

Aspartate aminotransferase (AST) and alanine aminotransferase (ALT) activities were measured using a commercially available kit (Transaminase CII test; Wako Pure Chemical Industries, Osaka, Japan) in plasma obtained from 15 mice per group.

### 2.3. Measurement of Hepatic Triglyceride Content

Pieces of liver were collected from 22-week-old fasting and fed mice (NCD, HFD, or HFD + MCDD); then, the hepatic triglyceride content was measured using a previously reported enzymatic assay method [19, 20].

### 2.4. Histopathological Examination

At 24 weeks of age, and after microCT scanning, mice were anesthetized with 2% isoflurane and transcardially perfused with 4% paraformaldehyde in 0.1 mol/l phosphate buffer solution (pH 7.2) (*n* = 10‐13 per group). Their livers were then dissected, fixed in 10% buffered formalin, embedded in paraffin, and sectioned at 3–4 *μ*m. The sections were stained with hematoxylin and eosin (H&E) or Sirius Red-Fast Green. WinROOF software (Mitani Corporation, Tokyo, Japan) was used to evaluate hepatic steatosis and fibrosis by image binarization processing. The histopathology was scored as follows: steatosis (0–3), lobular inflammation (0–3), and fibrosis (0–4), according to the grading and staging procedure for NASH [21], with minor modifications.

### 2.5. Analysis of Glucose and Insulin Homeostasis

The glucose and insulin tolerance of 6–8 mice per group were assessed before and after the 2 weeks of treatment. Intraperitoneal glucose tolerance testing (ipGTT) was performed in mice fasted overnight (16 hours) and injected intraperitoneally with glucose (1 g/kg body mass). Intraperitoneal insulin tolerance testing (ipITT) was performed after a 4 h fast, and involved the injection of human regular insulin (0.5 U/kg body mass, Eli Lilly and Company). Subsequently, blood glucose concentrations were determined using a glucometer (Medisafe, Terumo). Plasma insulin concentration was measured using an ELISA kit (Shibayagi, Gunma, Japan).

### 2.6. Evaluation of the Hemodynamics and Resolution of Ascites Using MicroCT

Blood pressure and heart rate were measured using the tail-cuff method (Model MK-2000ST, Muromachi Kikai Co. Ltd., Tokyo, Japan) just after the intraperitoneal infusion of MSA and also 5 hours later, at the end of the treatment period (*n* = 11‐15 mice per group). MSA was 10-fold diluted in PBS and was intraperitoneally infused at a volume of 0.1 ml/g body mass; then, microCT was performed hourly under inhalation anesthesia with 2% isoflurane (*n* = 6‐8 mice per group) to follow the changes in intra-abdominal fluid volume (CosmoScan GX scanner; Rigaku Corporation, Japan), as previously described [22]. The parameters used were as follows: 360° rotation, 360 projections, 1,700 ms exposure time, 90 kV voltage, 160 *μ*A current, and effective voxel size 148 × 148 × 148 *μ*m. Acquisitions were reconstructed using a filtered back-projection algorithm, a matrix size of 512 × 512 × 512 *μ*m, and the manufacturer's software.

### 2.7. Measurement of Plasma Hormone Concentration during Ascites Resolution

Plasma catecholamine (ImmuSmol, Pessac, France), renin (Thermo Fisher Scientific, MA, US), and aldosterone (Enzo, NY, US) concentrations were measured in the mice before and also 5 hours after the intraperitoneal injection of MSA (under nonstressed conditions after the induction of anesthesia using 2% isoflurane), using commercially available ELISA kits (*n* = 5‐7 mice per group).

### 2.8. Statistical Analyses

Data are presented as means ± standard errors of the means (SEMs). Statistical analysis was carried out using one-way analysis of variance (ANOVA), followed by the *post hoc* Bonferroni test. Pairs of groups were compared using the Wilcoxon matched-pairs test. All statistical analyses were performed using JMP for Windows (SAS Institute, Inc. Japan). Differences were considered significant when *P* < 0.05.

## 3. Results

### 3.1. Effect of HFD and MCDD Feeding and Each Treatment on Body Mass

Prior to drug administration, body mass gain was significantly more marked in mice fed an HFD for 17 weeks than in the NCD-fed group. However, in mice fed the HFD + MCDD, there was almost no body mass increase, and in fact their body mass was lower than that of the NCD-fed group. Two weeks of administration of dapagliflozin or furosemide reduced the body mass of the HFD-fed group, but no weight loss was induced in the NCD- or HFD + MCDD-fed groups by these agents (Figures 1(a)–1(c)).

### 3.2. Changes in Liver Enzyme Activities and Histological Findings

**Figure 2:**
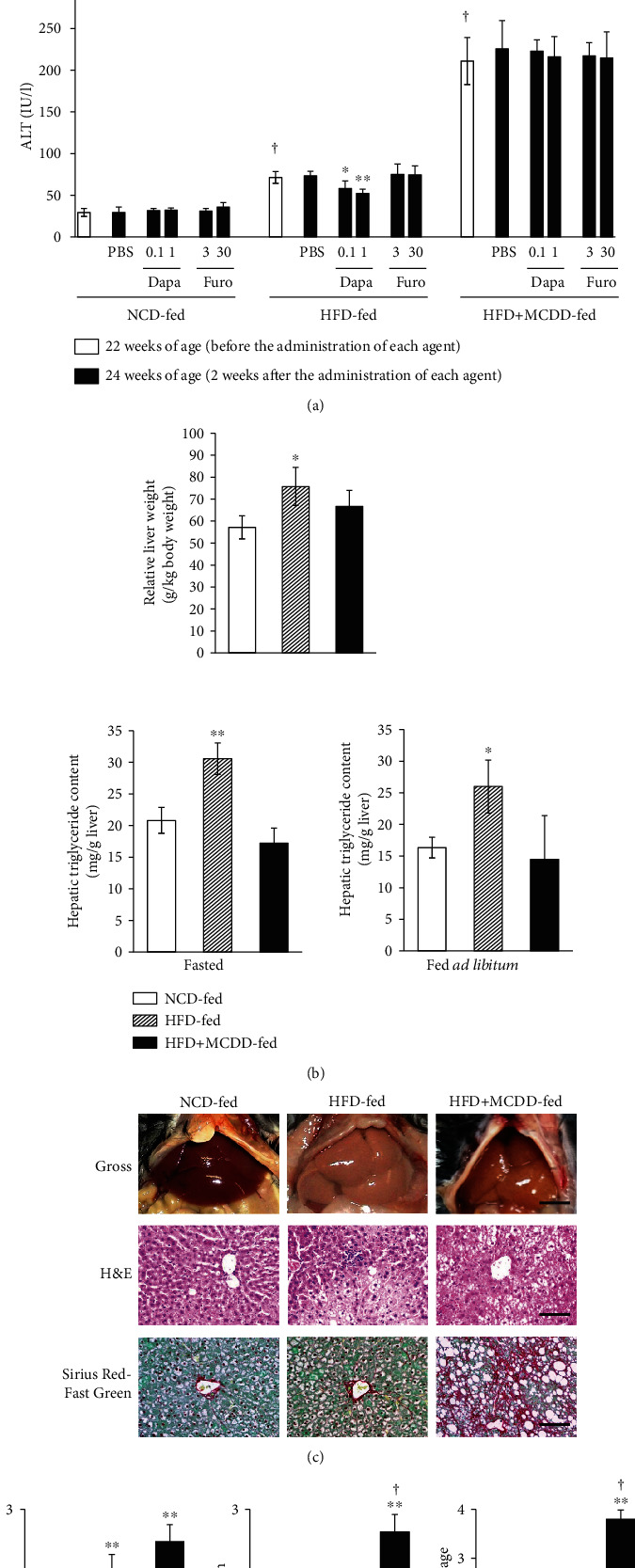
Blood transaminase activities were very high, and there was advanced liver fibrosis in MCDD-fed mice. (a) Plasma transaminase activities before and after 2 weeks of administration of each agent. *n* = 13‐15 per group. Data are means ± SEMs. ^†^*P* < 0.01 for the effect of diet, before the administration of each agent, by two-way ANOVA. ^∗^*P* < 0.05 and ^∗∗^*P* < 0.01*vs.* PBS in HFD-fed mice. (b) Relative liver mass (top; *n* = 6‐8 per group) and triglyceride content (bottom; *n* = 5‐7 per group) prior to treatment (NCD-, HFD-, or HFD + MCDD-fed). Data are means ± SEMs. ^∗^*P* < 0.05 and ^∗∗^*P* < 0.01 for the effect of diet by two-way ANOVA. (c) Gross appearance (top) and histopathology (H&E (middle) and Sirius Fast Red-Green (bottom) staining) of the livers of mice fed an NCD-, HFD-, or HFD + MCDD for 19 weeks. Scale bars: 5 mm (top) and 100 *μ*m (middle and bottom). (d) Score of steatosis, inflammation, and fibrosis stage. *n* = 10‐13 per group. Hepatic fibrosis was exacerbated by MCDD feeding. ^∗^*P* < 0.05 and ^∗∗^*P* < 0.01*vs.* NCD-fed mice. ^†^*P* < 0.01*vs.* HFD-fed mice.

Prior to treatment, the plasma transaminase activities in the HFD- and HFD + MCDD-fed groups were higher than those in the NCD-fed group. After 2 weeks of dapagliflozin treatment of HFD-fed mice, the plasma ALT, but not AST, activity was lower than in vehicle-treated mice, but the drug did not have a similar effect in HFD + MCDD-fed mice (Figure 2(a)). The livers of the HFD-fed group were pale, enlarged, and containing a high triglyceride concentration. In the HFD + MCDD-fed group, there was no marked difference in the color of the livers, but their surfaces were rough and their mean mass was lower (Figures 2(b) and 2(c)). Steatosis and inflammation were present in the livers of mice fed the HFD, and these pathological changes were worsened by methionine and choline deficiency. Sirius Red-Fast Green staining showed that liver fibrosis was marked in the HFD + MCDD-fed group (Figure 2(d)).

### 3.3. Fuel Homeostasis

**Figure 3:**
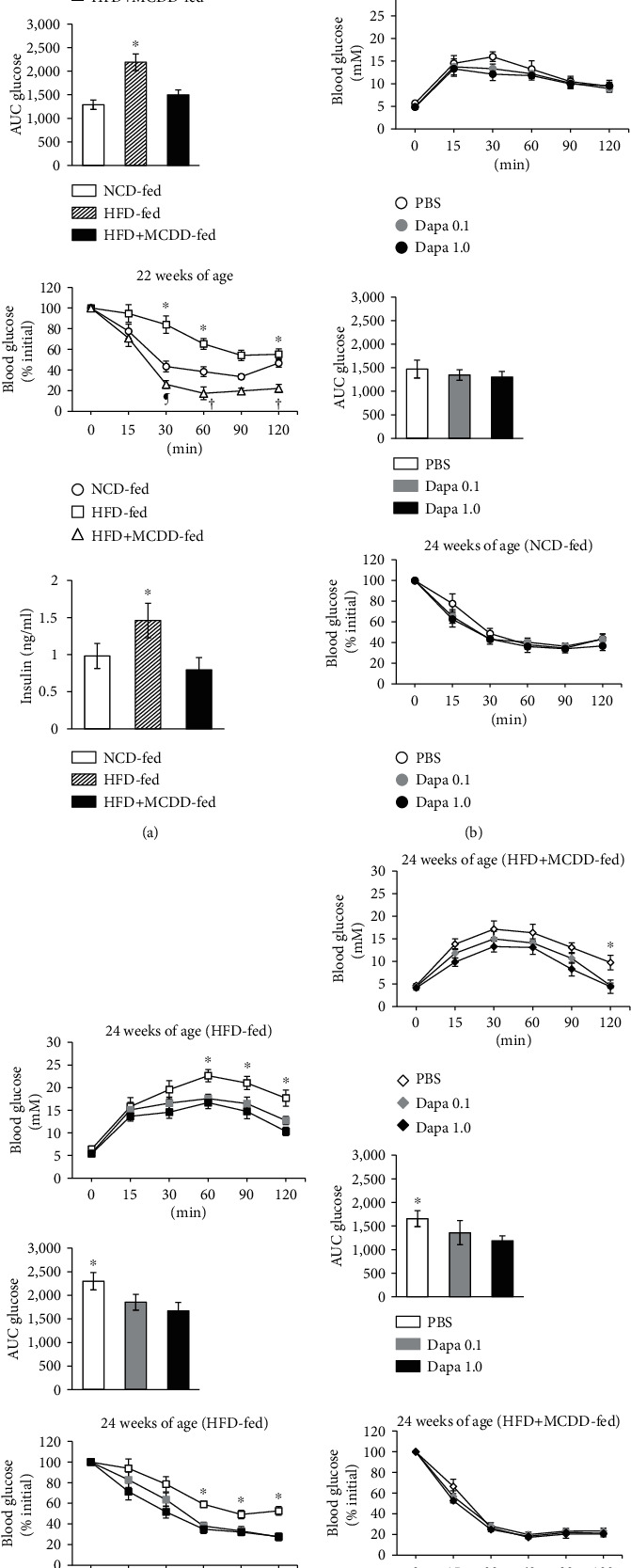
Glucose homeostasis in C57BL/6J mice consuming an MCDD is improved by the administration of dapagliflozin. (a) ipGTT in mice after 17 weeks of consumption of an NCD, HFD, or HFD + MCDD (top panel; *n* = 8 per group). The areas under the glucose curve (AUC glucose) for each group during the ipGTT are shown in the second panel. In addition, the glucose curve during an ipITT are shown in the third panel (*n* = 6‐8 per group). The plasma insulin concentrations of mice from each group fasted overnight for 16 hours (bottom panel; *n* = 8 per group). ^∗^*P* < 0.01 compared with the NCD- or HFD + MCDD-fed groups. ^†^*P* < 0.01 and ^¶^*P* < 0.05 compared with the NCD-fed group. (b) ipGTT glucose profiles (top panel) and AUC glucose (middle panel) for mice fed an NCD and treated with PBS, or 0.1 or 1.0 mg/kg/day dapagliflozin (Dapa 0.1 or 1.0) for 2 weeks (*n* = 8 per group). The ipITT glucose profiles are shown in the bottom panel (*n* = 6‐8 per group). (c) ipGTT glucose profiles (top panel) and AUC glucose (middle panel) in mice consuming an HFD that were treated with PBS, or 0.1 or 1.0 mg/kg/day dapagliflozin (Dapa 0.1 or 1.0) for 2 weeks (*n* = 7‐8 per group). The ipITT glucose profiles are shown in the bottom panel (*n* = 6‐8 per group). ^∗^*P* < 0.01*vs.* the Dapa 0.1 or 1.0-treated groups. (d) ipGTT glucose profiles (top panel) and AUC glucose (middle panel) in mice consuming an HFD + MCDD that were treated with PBS, or 0.1 or 1.0 mg/kg/day dapagliflozin (Dapa 0.1 or 1.0) for 2 weeks (*n* = 8 per group). The ipITT glucose profiles are shown in the bottom panel (*n* = 6‐8 per group). ^∗^*P* < 0.01 compared with the Dapa 1.0-treated group.

ipGTT showed that glucose tolerance was significantly poorer in the HFD-fed group than in the NCD-fed group prior to treatment. In addition, the glucose area under the curve (AUC) was significantly larger in the HFD + MCDD-fed group than in the NCD-fed group, despite the absence of weight gain. ipITT showed that insulin sensitivity was significantly lower in the HFD-fed group than in the NCD-fed group, and the fasting insulin concentration was higher. However, the insulin sensitivity of the HFD + MCDD-fed group was higher (Figure 3(a)). After 2 weeks of administration of dapagliflozin to HFD-fed mice, the glucose AUC during ipGTT was significantly lower and ipITT showed a significant improvement in insulin sensitivity. Dapagliflozin administration to the HFD + MCDD-fed group caused a significant, but smaller reduction in the glucose AUC during ipGTT and had no effect on insulin sensitivity (Figures 3(b)–3(d)).

### 3.4. Hemodynamics and Resolution of Ascites

The administration of dapagliflozin or furosemide for 2 weeks significantly reduced systolic blood pressure 5 hours later, regardless of the diet being consumed. However, dapagliflozin treatment did not affect diastolic blood pressure. Conversely, furosemide treatment caused reductions in diastolic blood pressure in all the diet groups. Furthermore, there was a significant increase in heart rate 5 hours after the administration of furosemide in each group, but there was no significant change in heart rate after dapagliflozin administration (Table 1).

**Figure 4:**
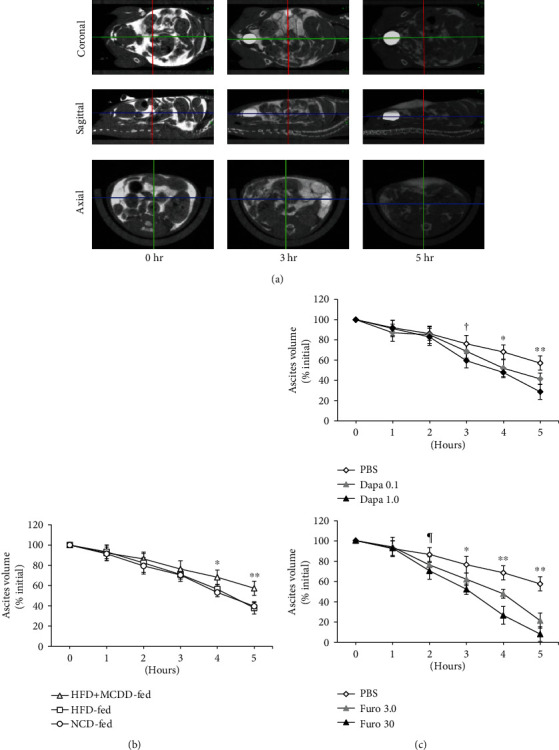
Fluid removal from the abdomen was poor in mice with liver cirrhosis, but was improved by dapagliflozin. (a) Representative views of the abdominal fluid over time (from left to right), obtained using microCT. Coronal (top), sagittal (middle), and axial (bottom) views. (b) Abdominal fluid volumes in mice fed each diet for 17 weeks (*n* = 8 per group). ^∗^*P* < 0.05 and ^∗∗^*P* < 0.01*vs.* the HFD- or NCD-fed groups. (c) Effects of each treatment on abdominal fluid removal in HFD + MCDD-fed mice. *n* = 6‐8 for each treatment group. ^∗^*P* < 0.05 and ^∗∗^*P* < 0.01*vs.* dapagliflozin- (top) or furosemide- (bottom) treated mice. ^†^*P* < 0.05*vs.* 1.0 mg/kg/day dapagliflozin-treated mice. ^¶^*P* < 0.05*vs.* 30 mg/kg/day furosemide-treated mice.

We next studied the kinetics of the loss of intraperitoneally infused MSA, which resembled ascites (Figure 4(a)). The fluid loss was slower in the HFD + MCDD-fed group than in the NCD or HFD-fed groups (Figure 4(b)), which suggests that ascites is likely to be retained in mice with liver cirrhosis induced by MCDD feeding. However, in HFD + MCDD-fed mice treated with dapagliflozin or furosemide, the rates of fluid removal were similar and significantly greater than in PBS-treated mice consuming the same diet (Figure 4(c)). However, furosemide simultaneously reduced the systolic and diastolic blood pressure, while increasing heart rate, whereas dapagliflozin did not affect these parameters.

### 3.5. Plasma Catecholamine, Renin, and Aldosterone Concentrations

**Figure 5:**
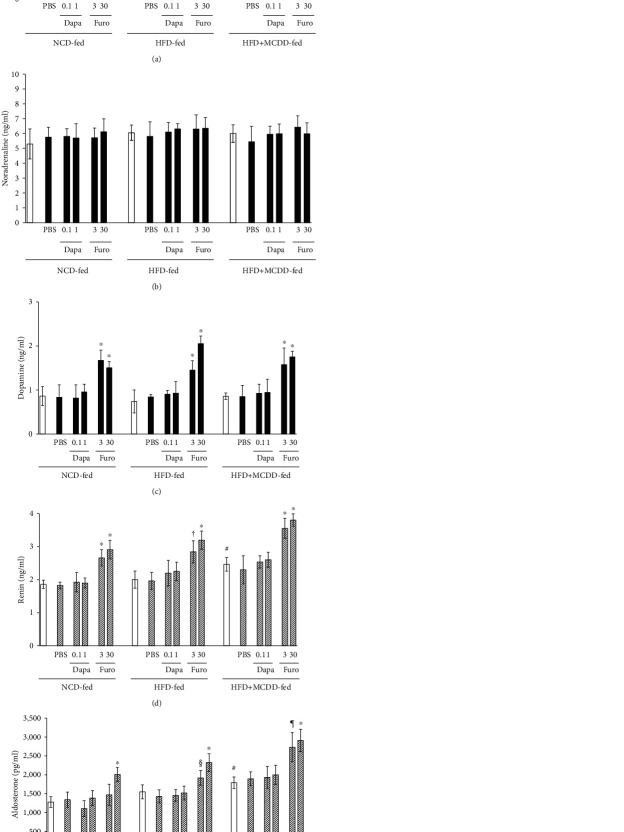
Dapagliflozin does not affect plasma catecholamine concentrations or the renin-aldosterone system (RAS). Plasma catecholamine (adrenaline (a), noradrenaline (b), and dopamine (c)) (closed bar) and RAS factor (renin (d) and aldosterone (e)) (hatched bar) concentrations before (open bar) and 5 hours after each treatment. *n* = 5‐7 for each treatment group. ^∗^*P* < 0.01*vs.* with PBS- or dapagliflozin-treated mice. ^†^*P* < 0.01*vs.* PBS-treated mice. ^§^*P* < 0.05*vs.* PBS- or dapagliflozin-treated mice. ^¶^*P* < 0.01*vs.* PBS- and *P* < 0.05*vs.* dapagliflozin-treated mice. ^#^*P* < 0.01*vs.* NCD-fed mice before each treatment.

Mice in all the diet groups had significantly higher plasma catecholamine concentrations (adrenaline and dopamine) 5 hours after furosemide administration. Five hours after dapagliflozin administration, the HFD + MCDD-fed group showed a trend toward higher plasma adrenaline concentration, but this did not reach significance (Figures 5(a)–5(c)). However, the plasma renin and aldosterone concentrations were higher in the HFD + MCDD-fed group than in the other groups. Furosemide, but not dapagliflozin, administration caused significant increases in all of these circulating factors 5 hours after administration (Figures 5(d) and 5(e)).

## 4. Discussion

The results of the present study suggest that dapagliflozin would be an effective treatment for patients with T2D, liver cirrhosis, and ascites. It might also be appropriate for the treatment of fatty liver, because it exhibited both glucose-lowering and ascites-resolving effects in HFD-fed mice, without having adverse effects on hemodynamics, even in the presence of liver fibrosis (Figures 2–4 and Table 1). Dapagliflozin slightly, but effectively reduced blood pressure, without influencing heart rate, in contrast to the more marked effects of furosemide, which has a potent diuretic effect. This implies that dapagliflozin does not affect catecholamine secretion or that it prevents any increase in heart rate by suppressing catecholamine action. In fact, we found no significant increase in circulating catecholamine concentrations in the dapagliflozin-treated group, in contrast to the effects of furosemide (Figures 5(a)–5(c)).

The mechanism by which dapagliflozin suppresses catecholamine secretion remains unclear. Furthermore, the detailed mechanism whereby ascites is resolved remains to be determined, but the present results are consistent with those of the EMPA-REG OUTCOME study [7], although the target organ differed. Empagliflozin markedly reduced the incidence of cardiovascular events, and the following mechanisms for this effect have been proposed: (1) a reduction of preload, secondary to osmotic diuresis; (2) a reduction in afterload, because of a reduction in body mass or blood pressure, or an increase in vascular dilatation; and (3) an inhibition of fatal arrhythmia, owing to the prevention of hypokalemia, secondary to a diuretic effect. However, these mechanisms may not be directly applicable to that mediating the resolution of ascites induced by dapagliflozin; hence, the mechanism of this effect must be studied in the future.

Unlike in the present study, Mirabelli et al. demonstrated that dapagliflozin was also effective at reducing diastolic pressure [23]. Although there was a similar trend in the present study, this was not significant. This discrepancy may be attributed to the fact that the mouse model of type 2 diabetes used in the present study was primarily one of NAFLD. Furthermore, because the study by Mirabelli et al. was conducted in humans, there could have been confounding effects of the concurrent administration of antihypertensive and/or lipid-lowering drugs. Thus, given the differences between the studies, it is difficult to directly compare the results of the present animal study with those of the previous human study.

In the presence of diabetes and fatty liver, liver function deteriorates, and as the condition progresses, the risk of NASH increases [24]. Le and Loomba reported higher values of indices of liver dysfunction in patients with NAFLD [25], but Kusunoki et al. showed that increases in AST and ALT activities could be reversed by treatment with a lipoprotein lipase-activating agent (NO-1886) that ameliorates fatty liver in a rat model of diabetes and fatty liver [26]. In addition, previous studies have shown that SGLT2i treatment ameliorates fatty liver in diabetic KKAy mice [27] and in a rat model of NASH induced by feeding a choline-deficient diet [28]. Honda et al. showed that administration of the SGLT2i ipragliflozin slows the pathogenesis of NASH by ameliorating insulin resistance and lipotoxicity in mice with NASH [29]. The randomized placebo-controlled trial EFFECT-II study demonstrated beneficial effects of the SGLT2i dapagliflozin on hepatocyte injury biomarkers, as well as plasma concentrations of fibroblast growth factor 21 (FGF21) in patients with NAFLD associated with T2D, which suggests reductions in cellular damage and fibrosis [30]. The dual SGLT1/2 inhibitor licogliflozin (Novartis) has also been reported to improve biomarkers and indices of liver function in a controlled trial, as reported at the AASLD annual meeting in 2019.

Several factors, such as the negative energy balance induced by the greater urinary glucose excretion, the inhibition of fat synthesis in the liver, and the increase in fat degradation, may be involved in the beneficial effects of SGLT2i on fatty liver [31]. However, there have been no studies to date regarding the efficacy of SGLT2i for the treatment of advanced liver cirrhosis with ascites. In addition, we have encountered patients with T2D and concomitant liver cancer or alcoholic liver injury. If the efficacy and safety of SGLT2i could be demonstrated for such patients and the indications for their use could be expanded, the risks associated with conventional insulin therapy could be avoided.

Recently, new drugs for the treatment of ascites have become commercially available, such as the vasopressin V2 receptor antagonist, tolvaptan. This drug does not reduce intravascular volume, in contrast to conventional loop diuretics, such as furosemide. As shown in Figures 5(d) and 5(e), the administration of dapagliflozin did not affect the circulating concentrations of components of the RAS, which is consistent with minimal effects on intravascular volume, diastolic blood pressure, and heart rate [32]. Therefore, the diuretic effect of dapagliflozin appears to be similar to that of tolvaptan. However, one previous study indicated that tolvaptan did not improve long-term prognosis [33]. Therefore, a long-term follow-up study of patients being treated with dapagliflozin should be performed. The higher circulating concentrations of RAS components in the HFD + MCDD-fed group, compared to the other diet groups, were associated with a reduction in intravascular volume (Figures 5(d) and 5(e)), which is likely to be an effect of the cirrhosis. We contend that this led to the observed delay in the spontaneous drainage of intra-abdominal fluid (Figure 4(b)).

A previous study of obese animals with T2D showed that SGLT2is increase urinary glucose excretion and reduce body mass [34]. In the present study, there was also a marked dapagliflozin-induced reduction in body mass in the HFD-fed group, but no such weight loss in the NCD- or HFD + MCDD-fed groups (Figures 1(b) and 1(c)). This may have been because the dapagliflozin administration period was short (2 weeks) and the MCDD itself may have caused weight loss in the HFD + MCDD-fed group. However, weight loss is not beneficial if it is accompanied by liver fibrosis, and glucose tolerance was poor in the HFD + MCDD-fed group (Figure 3). However, dapagliflozin administration ameliorated the glucose intolerance without increasing plasma transaminase activities, even in mice with liver cirrhosis induced by HFD + MCDD feeding, which suggests that dapagliflozin may be useful for the treatment of patients with T2D and liver cirrhosis (Figures 2(a) and 3(d)).

It has previously been shown that the circulating concentration of dapagliflozin is higher when the drug is administered to patients with severe hepatopathy. However, its half-life is shortened by the absence of enterohepatic circulation. In patients with moderate to severe hepatopathy, the half-life in the circulation is 6–8 hours, and dapagliflozin is excreted at a twofold higher rate than in other patients [35].

In patients with T2D and liver cirrhosis, insulin therapy often induces unexpected hypoglycemia. Liver fibrosis is associated with reductions in hepatic glycogen content and glycogenesis is affected; therefore, it is particularly important to ensure glycemic control and avoid hypoglycemia. Although there is a transient increase in the circulating concentration of dapagliflozin in patients with cirrhosis, the reduction in half-life may have contributed to the favorable outcomes with regard to the progression to liver fibrosis/cirrhosis in the present study.

Notably, when administering SGLT2is to patients with low insulin secretion who are not administering extrinsic insulin, serum ketone concentrations may increase or ketoacidosis may occur, and this is particularly true when administering these drugs to patients with liver cirrhosis. However, the results of a recent study suggested that ketone bodies might also be involved in the protective effects of SGLT2i [36], which implies that the role of ketone bodies should be further investigated.

Because the mechanism of action of SGLT2i is distinct from those of other antidiabetic drugs, they may be used as a monotherapy or in combination. A clinical trial (Dapagliflozin Efficacy and Action in NASH (DEAN)) by Huijie et al. (NCT03723252) is underway to assess the efficacy and safety of dapagliflozin for the treatment of NASH. However, their long-term safety in humans (especially for patients with cirrhosis and ascites) has not been verified; therefore, further longitudinal clinical studies are necessary.

There were several limitations to the present study. First, we were unable to evaluate urine volume, glucose and sodium concentrations, and osmotic pressure. Second, we did not attempt to replicate the findings using a genetically engineered animal model of T2D and NASH. Third, we were unable to quantify the expression of lipid metabolism genes, such as *Acc*, *Srebp1c*, *Cpt1a*, and *Ppparα*. Fourth, we did not assess intrinsic ascites because the volume was too small to be accurately evaluated using microCT. Finally, we did not characterize liver histology both before and after the administration of each agent; therefore, the effect of dapagliflozin on liver tissue is unclear. However, dapagliflozin administration reduced plasma transaminase activities in the HFD-fed group; therefore, it is likely that fatty liver may have improved or at least not progressed in these mice.

## 5. Conclusion

Dapagliflozin ameliorated the glucose intolerance and insulin resistance of both (fatty liver ± cirrhosis) mouse models, without inducing hypoglycemia. Furthermore, both drugs (dapagliflozin or furosemide) hastened the resolution of experimentally induced ascites, but unlike furosemide, dapagliflozin had no significant deleterious effects on hemodynamics, circulating catecholamine concentrations, or the renin-aldosterone axis. Finally, dapagliflozin reduced circulating transaminase activities in HFD + MCDD-fed mice, which suggests a reduction in ongoing liver damage. Therefore, it may be useful for the treatment of patients with both T2D and liver cirrhosis.

## Figures and Tables

**Table 1:** Effects of each agent on hemodynamic parameters.

		Baseline	PBS group		Dapa 0.1 group		Dapa 1.0 group		Furo 3.0 group		Furo 30 group	
			5 hours after	*P* value	5 hours after	*P* value	5 hours after	*P* value	5 hours after	*P* value	5 hours after	*P* value
NCD-fed	SBP (mmHg)	109.1 ± 3.4	108.3 ± 3.3	0.868	107.4 ± 3.1	0.497	100.7 ± 3.3	0.028	99.0 ± 4.0	0.063	92.9 ± 3.0	0.000
	DBP (mmHg)	73.7 ± 1.9	72.7 ± 2.0	0.714	72.2 ± 1.8	0.647	71.5 ± 2.0	0.530	69.3 ± 1.4	0.053	66.7 ± 2.1	0.007
	HR (bpm)	542.9 ± 13.8	548.9 ± 12.2	0.747	542.3 ± 11.9	0.719	551.8 ± 16.6	0.498	603.5 ± 13.7	0.001	592.9 ± 20.5	0.027
HFD-fed	SBP (mmHg)	122.6 ± 2.9	121.2 ± 2.5	0.715	113.5 ± 3.0	0.070	106.7 ± 4.0	0.011	108.7 ± 4.7	0.024	109.2 ± 3.7	0.010
	DBP (mmHg)	74.3 ± 1.4	74.3 ± 1.0	0.975	73.6 ± 1.1	0.683	72.7 ± 1.4	0.379	67.5 ± 1.7	0.003	63.9 ± 1.9	0.000
	HR (bpm)	536.2 ± 16.4	529.5 ± 15.7	0.771	542.4 ± 17.6	0.768	554.6 ± 11.1	0.322	559.9 ± 13.8	0.266	584.7 ± 15.5	0.041
HFD+ MCDD-fed	SBP (mmHg)	107.5 ± 3.0	106.4 ± 2.3	0.986	100.9 ± 2.9	0.249	94.5 ± 3.4	0.010	95.7 ± 3.6	0.042	86.8 ± 4.2	0.001
	DBP (mmHg)	72.1 ± 1.3	72.2 ± 1.3	0.943	71.3 ± 2.1	0.759	69.7 ± 1.2	0.471	64.3 ± 2.0	0.010	63.8 ± 2.4	0.012
	HR (bpm)	535.1 ± 15.5	545.2 ± 15.0	0.644	542.7 ± 12.8	0.758	544.9 ± 16.3	0.664	583.3 ± 17.2	0.045	578.7 ± 12.5	0.007

NCD = normal chow diet; HFD = high-fat diet; MCDD = methionine-and-choline-deficient diet; SBP = systolic blood pressure; DBP = diastolic blood pressure; HR = heart rate; PBS = phosphate-buffered saline; Dapa 0.1 = administration of dapagliflozin 0.1 mg/kg/day for 2 weeks; Dapa 1.0 = administration of dapagliflozin 1.0 mg/kg/day for 2 weeks; Furo 3.0 = administration of furosemide 3.0 mg/kg/day for 2 weeks; Furo 30 = administration of furosemide 30 mg/kg/day for 2 weeks. Blood pressure and heart rate immediately after intraperitoneal injection of MSA and 5 hours later are shown (*n* = 11‐15). Differences between baseline (0 hours) and the 5-hour time point were analyzed using the Wilcoxon matched-pairs signed-rank test.

## Data Availability

The data used to support the findings of the present study are shown in the tables and figures of the article.
